# Molecular neuroimaging in dominantly inherited versus sporadic early-onset Alzheimer’s disease

**DOI:** 10.1093/braincomms/fcae159

**Published:** 2024-05-03

**Authors:** Leonardo Iaccarino, Jorge J Llibre-Guerra, Eric McDade, Lauren Edwards, Brian Gordon, Tammie Benzinger, Jason Hassenstab, Joel H Kramer, Yan Li, Bruce L Miller, Zachary Miller, John C Morris, Nidhi Mundada, Richard J Perrin, Howard J Rosen, David Soleimani-Meigooni, Amelia Strom, Elena Tsoy, Guoqiao Wang, Chengjie Xiong, Ricardo Allegri, Patricio Chrem, Silvia Vazquez, Sarah B Berman, Jasmeer Chhatwal, Colin L Masters, Martin R Farlow, Mathias Jucker, Johannes Levin, Stephen Salloway, Nick C Fox, Gregory S Day, Maria Luisa Gorno-Tempini, Adam L Boxer, Renaud La Joie, Randall Bateman, Gil D Rabinovici

**Affiliations:** Memory and Aging Center, Department of Neurology, Weill Institute for Neurosciences, University of California San Francisco, San Francisco, CA 94158, USA; The Dominantly Inherited Alzheimer Network (DIAN), St Louis, MO 63108, USA; Department of Neurology, Washington University in St Louis, St Louis, MO 63108, USA; The Dominantly Inherited Alzheimer Network (DIAN), St Louis, MO 63108, USA; Department of Neurology, Washington University in St Louis, St Louis, MO 63108, USA; Memory and Aging Center, Department of Neurology, Weill Institute for Neurosciences, University of California San Francisco, San Francisco, CA 94158, USA; Department of Radiology, Washington University in St Louis, St Louis, MO 63110, USA; Department of Radiology, Washington University in St Louis, St Louis, MO 63110, USA; The Dominantly Inherited Alzheimer Network (DIAN), St Louis, MO 63108, USA; Department of Neurology, Washington University in St Louis, St Louis, MO 63108, USA; Memory and Aging Center, Department of Neurology, Weill Institute for Neurosciences, University of California San Francisco, San Francisco, CA 94158, USA; Department of Biostatistics, Washington University in St Louis, St Louis, MO 63110, USA; Memory and Aging Center, Department of Neurology, Weill Institute for Neurosciences, University of California San Francisco, San Francisco, CA 94158, USA; Memory and Aging Center, Department of Neurology, Weill Institute for Neurosciences, University of California San Francisco, San Francisco, CA 94158, USA; The Dominantly Inherited Alzheimer Network (DIAN), St Louis, MO 63108, USA; Department of Neurology, Washington University in St Louis, St Louis, MO 63108, USA; Memory and Aging Center, Department of Neurology, Weill Institute for Neurosciences, University of California San Francisco, San Francisco, CA 94158, USA; Department of Pathology and Immunology, Washington University in St Louis, St Louis, MO 63110, USA; Memory and Aging Center, Department of Neurology, Weill Institute for Neurosciences, University of California San Francisco, San Francisco, CA 94158, USA; Memory and Aging Center, Department of Neurology, Weill Institute for Neurosciences, University of California San Francisco, San Francisco, CA 94158, USA; Memory and Aging Center, Department of Neurology, Weill Institute for Neurosciences, University of California San Francisco, San Francisco, CA 94158, USA; Memory and Aging Center, Department of Neurology, Weill Institute for Neurosciences, University of California San Francisco, San Francisco, CA 94158, USA; Department of Biostatistics, Washington University in St Louis, St Louis, MO 63110, USA; Department of Biostatistics, Washington University in St Louis, St Louis, MO 63110, USA; Department of Cognitive Neurology, Institute for Neurological Research Fleni, Buenos Aires 1428, Argentina; Department of Cognitive Neurology, Institute for Neurological Research Fleni, Buenos Aires 1428, Argentina; Department of Cognitive Neurology, Institute for Neurological Research Fleni, Buenos Aires 1428, Argentina; Department of Neurology, University of Pittsburgh, Pittsburgh, PA 15213, USA; Massachusetts General Hospital, Harvard Medical School, Boston, MA 02114, USA; Department of Neuroscience, Florey Institute, The University of Melbourne, Melbourne 3052, Australia; Neuroscience Center, Indiana University School of Medicine at Indianapolis, Indiana, IN 46202, USA; DZNE-German Center for Neurodegenerative Diseases, Tübingen 72076, Germany; Department of Neurology, Ludwig-Maximilians-University, Munich 80539, Germany; German Center for Neurodegenerative Diseases, Munich 81377, Germany; Munich Cluster for Systems Neurology (SyNergy), Munich 81377, Germany; Memory & Aging Program, Butler Hospital, Brown University in Providence, RI 02906, USA; Dementia Research Centre, Department of Neurodegenerative Disease, University College London Institute of Neurology, London WC1N 3BG, UK; Department of Neurology, Mayo Clinic Florida, Jacksonville, FL 33224, USA; Memory and Aging Center, Department of Neurology, Weill Institute for Neurosciences, University of California San Francisco, San Francisco, CA 94158, USA; Memory and Aging Center, Department of Neurology, Weill Institute for Neurosciences, University of California San Francisco, San Francisco, CA 94158, USA; Memory and Aging Center, Department of Neurology, Weill Institute for Neurosciences, University of California San Francisco, San Francisco, CA 94158, USA; The Dominantly Inherited Alzheimer Network (DIAN), St Louis, MO 63108, USA; Department of Neurology, Washington University in St Louis, St Louis, MO 63108, USA; Memory and Aging Center, Department of Neurology, Weill Institute for Neurosciences, University of California San Francisco, San Francisco, CA 94158, USA; Department of Radiology and Biomedical Imaging, University of California, San Francisco, CA 94143, USA

**Keywords:** Alzheimer’s disease, amyloid-PET, brain glucose metabolism, FDG-PET

## Abstract

Approximately 5% of Alzheimer’s disease patients develop symptoms before age 65 (early-onset Alzheimer’s disease), with either sporadic (sporadic early-onset Alzheimer’s disease) or dominantly inherited (dominantly inherited Alzheimer’s disease) presentations. Both sporadic early-onset Alzheimer’s disease and dominantly inherited Alzheimer’s disease are characterized by brain amyloid-β accumulation, tau tangles, hypometabolism and neurodegeneration, but differences in topography and magnitude of these pathological changes are not fully elucidated. In this study, we directly compared patterns of amyloid-β plaque deposition and glucose hypometabolism in sporadic early-onset Alzheimer’s disease and dominantly inherited Alzheimer’s disease individuals. Our analysis included 134 symptomatic sporadic early-onset Alzheimer’s disease amyloid-Positron Emission Tomography (PET)-positive cases from the University of California, San Francisco, Alzheimer’s Disease Research Center (mean ± SD age 59.7 ± 5.6 years), 89 symptomatic dominantly inherited Alzheimer’s disease cases (age 45.8 ± 9.3 years) and 102 cognitively unimpaired non-mutation carriers from the Dominantly Inherited Alzheimer Network study (age 44.9 ± 9.2). Each group underwent clinical and cognitive examinations, ^11^C-labelled Pittsburgh Compound B-PET and structural MRI. ^18^F-Fluorodeoxyglucose-PET was also available for most participants. Positron Emission Tomography scans from both studies were uniformly processed to obtain a standardized uptake value ratio (PIB_50–70_ cerebellar grey reference and FDG_30–60_ pons reference) images. Statistical analyses included pairwise global and voxelwise group comparisons and group-independent component analyses. Analyses were performed also adjusting for covariates including age, sex, Mini-Mental State Examination, apolipoprotein ε4 status and average composite cortical of standardized uptake value ratio. Compared with dominantly inherited Alzheimer’s disease, sporadic early-onset Alzheimer’s disease participants were older at age of onset (mean ± SD, 54.8 ± 8.2 versus 41.9 ± 8.2, Cohen’s *d* = 1.91), with more years of education (16.4 ± 2.8 versus 13.5 ± 3.2, *d* = 1) and more likely to be apolipoprotein ε4 carriers (54.6% ε4 versus 28.1%, Cramer’s *V* = 0.26), but similar Mini-Mental State Examination (20.6 ± 6.1 versus 21.2 ± 7.4, *d* = 0.08). Sporadic early-onset Alzheimer’s disease had higher global cortical Pittsburgh Compound B-PET binding (mean ± SD standardized uptake value ratio, 1.92 ± 0.29 versus 1.58 ± 0.44, *d* = 0.96) and greater global cortical ^18^F-fluorodeoxyglucose-PET hypometabolism (mean ± SD standardized uptake value ratio, 1.32 ± 0.1 versus 1.39 ± 0.19, *d* = 0.48) compared with dominantly inherited Alzheimer’s disease. Fully adjusted comparisons demonstrated relatively higher Pittsburgh Compound B-PET standardized uptake value ratio in the medial occipital, thalami, basal ganglia and medial/dorsal frontal regions in dominantly inherited Alzheimer’s disease versus sporadic early-onset Alzheimer’s disease. Sporadic early-onset Alzheimer’s disease showed relatively greater ^18^F-fluorodeoxyglucose-PET hypometabolism in Alzheimer’s disease signature temporoparietal regions and caudate nuclei, whereas dominantly inherited Alzheimer’s disease showed relatively greater hypometabolism in frontal white matter and pericentral regions. Independent component analyses largely replicated these findings by highlighting common and unique Pittsburgh Compound B-PET and ^18^F-fluorodeoxyglucose-PET binding patterns. In summary, our findings suggest both common and distinct patterns of amyloid and glucose hypometabolism in sporadic and dominantly inherited early-onset Alzheimer’s disease.

## Teaser

Iaccarino *et al*. report that sporadic early-onset Alzheimer’s disease and dominantly inherited early-onset Alzheimer’s disease show both common and distinct patterns of amyloid pathology and neurodegeneration. These findings highlight overlapping pathophysiological features across aetiologies, capturing some heterogeneity in biomarker findings potentially due to varying properties of pathological aggregates and variant-specific regional vulnerabilities.

## Graphical Abstract

**Graphical abstract:**
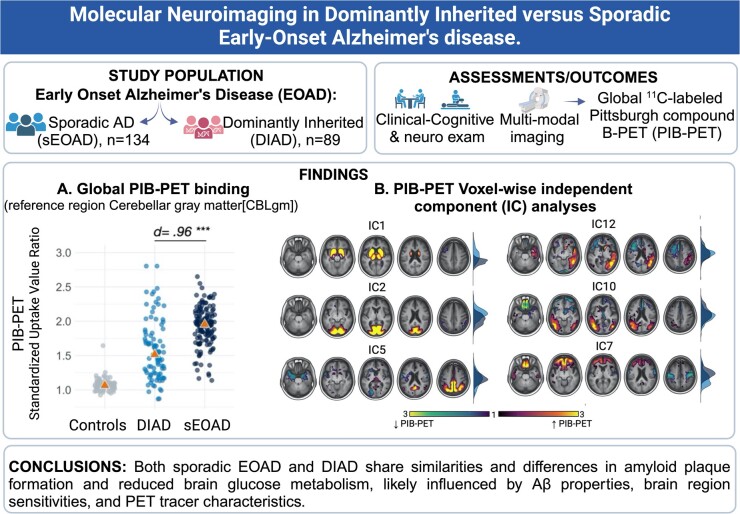

## Introduction

Alzheimer’s disease is the most common cause of dementia worldwide^1^ and is biologically defined by pathological accumulation of amyloid-β (Aβ) plaques and neurofibrillary tau tangles.^2^ Alzheimer’s disease can be either sporadic or due to dominantly inherited mutations in the genes *Presinilin-1* (*PSEN1*), *PSEN2* or *Amyloid Precursor Protein* (*APP*). The majority of individuals with Alzheimer’s disease develop symptoms in older age in the absence of a pathogenic gene mutation [sporadic late-onset Alzheimer’s disease (LOAD)^3,4^]. Approximately 5% of patients develop symptoms before age 65 [early-onset Alzheimer’s disease (EOAD)]^5^—of these, approximately 5–10% have a dominantly inherited form of Alzheimer’s disease (DIAD, most commonly due to mutations in *PSEN1*), and the rest have apparently sporadic EOAD (sEOAD^6,7^).^7-9^

Clinical studies comparing DIAD versus sporadic LOAD have found similar cognitive syndromes, although with more rapid cognitive decline^3^ and a higher prevalence of motor manifestations (e.g. myoclonus and spasticity)^10-12^ in DIAD. Biomarker studies have demonstrated that DIAD and sporadic LOAD may be associated with different Aβ species (lower CSF Aβ_37_, Aβ_38_ and Aβ_39_ in DIAD),^13^ with DIAD patients also showing higher cortical tau-PET binding,^14,15^ more severe neurodegeneration in Alzheimer’s disease signature and other regions^16-19^ and more advanced neuropathology at autopsy.^20-25^ As for amyloid-Positron Emission Tomography (PET) measures, these have been suggested to be largely comparable between DIAD and LOAD patients, especially in neocortical regions, with some evidence for relatively higher binding in basal ganglia in DIAD.^26^ Comparisons of sporadic forms of Alzheimer’s disease by age of onset similarly revealed accelerated cognitive decline,^27,28^ differential Aβ species accumulation (lower CSF Aβ_43_),^29^ higher cortical tau-PET binding,^30-35^ more severe neurodegeneration^34,36-42^ and more advanced pathology at autopsy in sEOAD compared with sporadic LOAD.^43-46^ As for amyloid-PET measures, previous studies have overall demonstrated null or weak associations between magnitude and/or spatial extent of binding elevation and age in sporadic Alzheimer’s disease.^34,35^ The observation that DIAD and sEOAD share a younger age and similar clinical and biomarker profiles compared with sporadic LOAD is of particular importance, suggesting complex relationships among Alzheimer’s disease pathology, age and genetic status.

These studies overall seem to suggest that clinical and biomarker trajectories, within the Alzheimer’s disease pathophysiological cascade, may be modulated by age, both in sporadic and in DIAD patients. Still, there is limited evidence comparing imaging biomarkers among sEOAD and DIAD groups, potentially more directly uncovering Alzheimer’s disease pathophysiological differences less related to age and more likely to be associated to genetic status. In this study, we directly compared a sEOAD cohort followed at the University of California San Francisco Alzheimer’s Disease Research Center (UCSF ADRC) and a cohort of DIAD individuals from the multi-site Dominantly Inherited Alzheimer Network (DIAN). Within this collaborative study, we systematically compared available PET imaging biomarkers [i.e. ^11^C-Pittsburgh Compound B (PIB)-PET (PIB-PET, targeting Aβ accumulation) and ^18^F-fluorodeoxyglucose (^18^F-FDG)-PET (FDG-PET, targeting brain glucose metabolism)]. A complementary comparison of clinical and cognitive measures and fluid biomarkers is reported in a separate manuscript.^47^ We aimed to (i) compare global imaging metrics and voxel-level PIB-PET and FDG-PET binding in sEOAD versus DIAD and (ii) identify independent PIB-PET and FDG-PET binding patterns in symptomatic participants and compare their expression in the two groups.

## Materials and methods

### Participants

#### Inclusion criteria

##### sEOAD cohort

sEOAD participants from the UCSF ADRC were required to (i) have an available and positive PIB-PET scan to establish Alzheimer’s continuum,^48^ (ii) have undergone a detailed neurological and neuropsychological examination, (iii) be symptomatic [Clinical Dementia Rating (CDR®) total score ≥ 0.5] at PIB-PET time, (iv) have age at reported symptoms onset < 65 years old, (v) have absence of a family history of dementia that followed an autosomal dominant pattern and did not have evidence of a mutation associated with DIAD and (vi) have a clinical diagnosis of MCI or dementia due to Alzheimer’s disease.^49,50^ PIB-PET positivity in the sEOAD group was assessed both at visual read and quantitation as previously described.^51^

##### DIAD cohort

DIAD participants were required to (i) have an available PIB-PET scan, (ii) have undergone a detailed neurological and neuropsychological examination, (iii) be symptomatic (CDR® total score ≥ 0.5) at PIB-PET time, (iv) have age at reported symptoms onset < 65 years old and (v) carry a known dominantly inherited pathogenic mutation in *PSEN1*, *PSEN2* or *APP* to establish Alzheimer’s disease. Detailed inclusion criteria for the parent DIAN study are available at https://dian.wustl.edu.

##### CN cohort

Cognitively unimpaired (global CDR = 0) non-mutation carriers DIAN participants with an available PIB-PET scan were included in the present study as a cognitively normal (CN) control. CN controls underwent the same diagnostic procedures as DIAD participants.

All participants had available demographics, neurological and neuropsychological examinations. For DIAD participants, PIB-PET, FDG-PET and structural MRI were acquired at the same visit of the clinical and cognitive assessment (DIAD). For sEOAD participants, PIB-PET, FDG-PET and structural MRI were acquired within 1 year from the clinical and cognitive assessment. FDG-PET was available for ∼88% of the participants, and apolipoprotein E (*APOE*) genotyping data were also available for ∼99% of participants (see below and Table 1).

**Table 1:** Demographic, clinical and biomarker summary

Group	CN	sEOAD	DIAD	Effect size	*P*_FDR_
Cohort	DIAN	UCSF	DIAN	-	-
Sample size—*N*	102	134	89	-	-
Age at PET (years)	44.9 (9.2)	59.7 (5.6)	45.8 (9.3)	1.91	**<0.001**
Age at onset (years)	-	54.8 (5.3)	41.9 (8.2)	1.96	**<0**.**001**
Sex—*N* (%) female	60 (58.8%)	76 (56.7%)	53 (59.5%)	0.03	0.8
Symptom duration (years)	-	4.9 (2.8)	4.1 (3.5)	0.26	0.09
Education (years)	14.6 (2.6)	16.4 (2.8)	13.5 (3.2)	1	**<0**.**001**
CDR-SB	0.03 (0.18)	4.48 (2.26)	5.73 (5.5)	0.32	0.06
MMSE	29.2 (1.1)	20.6 (6.1)	21.2 (7.4)	0.08	0.6
Global cortical PIB-PET SUVR	1.07 (0.08)	1.92 (0.29)	1.58 (0.44)	0.96	**<0**.**001**
PIB-PET Centiloid values	−3 (9)	94 (32)	55 (50)	0.96	**<0**.**001**
Global cortical FDG-PET SUVR	1.56 (0.13)	1.32 (0.1)	1.39 (0.19)	0.48	**0**.**002**
CDR total score | *N* 0/0.5/1/2/3	102/0/0/0/0	0/61/68/2/1	0/51/20/15/3	0.37	**<0**.**001**
APOE ε4 Status—*N* (%) carrier	35 (34.3%)	71 (54.6%)	25 (28.1%)	0.26	**<0**.**001**
DIAD gene | *PSEN1/PSEN2/APP*	-	-	75/2/12	-	-
FDG-PET Available—*N*(%)	87 (85.3%)	113 (84.3%)	88 (98.9%)	0.24	**0**.**001**

Table shows demographic, clinical and biomarker summary split by cohorts. Effect sizes and *P*-values refer to sEOAD versus DIAD comparisons, values displayed in bold indicate statistically significant differences. Continuous variables are presented as mean (SD). *P*-values are corrected with a FDR correction for multiple comparisons. Effect sizes were computed as Cohen’s *d* for continuous variables and Cramer’s *V* for discrete variables comparisons.

Missing data: CDR total/sb was missing for *N* = 2 sEOAD participants. APOE ε4 Status was missing for *N* = 4 sEOAD participants. Education years info was missing for *N* = 26 CN, *N* = 13 DIAD and *N* = 1 sEOAD participants. MMSE was missing for *N* = 1 CN and *N* = 2 sEOAD participants. FDG-PET data were missing for *N* = 15 CN, *N* = 1 DIAD and *N* = 21 sEOAD participants.

CN, cognitively normal; sEOAD, sporadic early-onset Alzheimer’s disease; DIAD, dominantly inherited Alzheimer’s disease; FDR, false discovery rate; DIAN, Dominantly Inherited Alzheimer Network; UCSF, University of California San Francisco; CDR sb, Clinical Dementia Rating sum of boxes; MMSE, Mini-Mental State Examination; APOE, apolipoprotein E; PSEN1, Presinilin-1; PSEN2, Presinilin-2; APP, Amyloid Precursor Protein.

### Data selection

#### sEOAD cohort

Data querying (March 2019) identified *N* = 134 sEOAD participants meeting inclusion criteria, all recruited from the UCSF ADRC between 2005 and 2019. Two participants did not have CDR available but had Mini-Mental State Examination (MMSE) scores < 24 and were thus included as symptomatic. FDG-PET scans were available for 113/134 (∼84%) sEOAD. All the PIB-PET scans included for the sEOAD cohort were baseline scans.

#### DIAD and CN cohorts

Data from DIAN Data Freeze 13 were included. For this analysis, we included DIAD symptomatic participants and asymptomatic non-mutation carriers as controls. Querying for participants with a complete PIB-PET scan passing quality control, we found *N* = 326 visits belonging to *N* = 211 (*N* = 110/211 non-mutation carriers and *N* = 101/211 mutation carriers) unique participants. As DIAN participants were on average younger than sEOAD participants, we selected the latest PIB-PET time point available for the *N* = 211 participants, i.e. selecting the last visit in case multiple visits were available. Considering the last visits, non-mutation carriers with a CDR total score of >0 (*N* = 8) and mutation carriers with a CDR total score of 0 (*N* = 12) were excluded. This resulted in a total of *N* = 89 symptomatic mutation carriers (DIAD group) and *N* = 102 CN non-mutation carriers (CN group). FDG-PET scans were available for 88/89 (∼99%) DIAD and 87/102 (∼85%) CN participants. There were no mutation carriers with the APP E693Q variant (Dutch type CAA), which has previously been shown to demonstrate minimal PIB-PET abnormalities and a different clinical progression.

### MRI acquisition

#### sEOAD cohort

Structural MRIs were acquired either at the UCSF Neuroimaging Center (*N* = 109/134) or at the San Francisco Veterans Affairs Medical Centre (*N* = 25/134) as high-resolution T_1_-weighted magnetization-prepared rapid gradient echo sequences. UCSF Neuroimaging Center scans were performed on either a 3T Siemens Tim Trio scanner (*N* = 61) or on a 3T Siemens Prisma Fit (*N* = 48). San Francisco Veterans Affairs Medical Centre scans were performed on either a 1.5T Siemens VISION system (*N* = 17) or on a 4T Bruker MedSpec system controlled by a Siemens Trio console (*N* = 8). Specific acquisition parameters have been outlined previously.^34,52,53^

#### DIAD and CN cohorts

Details on study procedures are available on the DIAN website (https://dian.wustl.edu/). All MRI scans were performed on validated scanners by the participating sites and included a T_1_-weighted magnetization-prepared rapid gradient echo scan that was used for further processing in the present study.

### MRI processing

All structural MRIs used in the present study (regardless of cohort and/or scanner) underwent the same processing pipeline at UCSF, including rigid-body co-registration to a template MRI and processing with Freesurfer 7.1 (http://surfer.nmr.mgh.harvard.edu/), additionally using the brainstem substructures parcellation module.^54^ MRIs were then segmented with SPM12 (https://www.fil.ion.ucl.ac.uk/spm/software/spm12/) saving forward deformation parameters. Quality control was performed on all MRI processing files to ensure correct parcellation and segmentation. While the FDG-PET acquisitions were more compatible between UCSF and DIAN (see below), the structural MRI acquisitions were largely heterogeneous across participants and cohorts and were thus not considered for further statistical analyses but only used to process PET scans (see below).

### PET acquisition

#### sEOAD cohort

PIB-PET and FDG-PET were acquired at the Lawrence Berkeley National Laboratory (Berkeley, CA, USA) with either a Siemens ECAT EXACT HR (*N* = 45 FDG-PET; *N* = 47 PIB-PET) or a Siemens Biograph 6 Truepoint PET/CT scanner in 3D acquisition mode (*N* = 68 FDG-PET; *N* = 87 PIB-PET). Attenuation correction was performed using a low-dose CT/transmission scan acquired prior to all the PET scans. For PIB-PET, dynamic acquisition was performed for 90 min (35 frames total) immediately after intravenous injection of ∼15 mCi of ^11^C-PiB. For FDG-PET, a 30-min scan (6 × 5 min frames) was acquired 30 min following i.v. injection of 5–10 mCi of ^18^F-FDG (resting quietly with eyes open during acquisition). All images were acquired in list mode and reconstructed using an ordered subset expectation maximum algorithm with weighted attenuation. Biograph and ECAT images were smoothed using a 4-mm Gaussian kernel with scatter correction during reconstruction (calculated image resolution Biograph 6.5 × 6.5 × 7.25 mm, ECAT 7 × 7 × 7.5 mm using Hoffman phantom).

#### DIAD and CN cohorts

Details on study procedures are available on the DIAN website (https://dian.wustl.edu/). Available dynamic PIB-PET and FDG-PET scans were downloaded in their original format for further processing. For both tracers, only data with the expected acquisition protocol were included for further processing. For PIB-PET, this included a 33-frame, 70-min-long dynamic scan starting at time of injection or a 6-frame, 30-min-long dynamic scan starting 40 min post-injection of 8–18 mCi (min-max) of ^11^C-PiB. For FDG-PET, the scan was performed as a 6-frame, 30-min-long acquisition starting 30 min post-injection of 5 ± 0.5 mCi of ^18^F-FDG.

### PET pre-processing

PET data from UCSF and DIAN cohorts underwent the same pre-processing pipeline at UCSF regardless of cohort and/or scanner. Frames representing 50–70 min post-injection for PIB-PET and 30–60 min post-injection for FDG-PET were selected, rigidly realigned and averaged. A differential smoothing approach was then applied to bring all the PET images to an estimated 8-mm^3^ resolution, using scanner-specific values as provided in DIAN for the DIAD cohort and as calculated for the sEOAD cohort based on Hoffman phantom acquisitions. Reference regions were defined via Freesurfer and the brainstem parcellation module to identify cerebellar grey matter (for PIB-PET) and pons (for FDG-PET). Averaged, smoothed PET scans were co-registered to their respective structural MRIs and intensity normalized according to average reference region binding, generating standardized uptake value ratio (SUVR) images for both PIB-PET (SUVR_50–70_) and FDG-PET (SUVR_30–60_) images. PET SUVR images were warped to the Montreal Neurological Institute space using the deformation parameters generated by the SPM12 segmentation of the respective structural MRI. For voxelwise analysis, an explicit mask was generated by averaging whole group warped grey and white matter segmentations, thresholding the image at >0.2 and manually removing the brainstem and cerebellum.

### PET analysis

#### Global binding group-level comparisons

First, a summary average PIB-PET (cerebellar grey reference) and FDG-PET (pons reference) SUVR was estimated including all cortical regions (Desikan–Killiany atlas, https://surfer.nmr.mgh.harvard.edu/fswiki/CorticalParcellation) as defined by Freesurfer. We then tested additional combinations of target regions in order to investigate their relative impact on sEOAD versus DIAD comparisons. We first estimated average binding in a more restricted neocortical target region including frontal, temporal and parietal regions, using cerebellar grey as a reference, which has been our default approach to investigate binding in Alzheimer’s disease-related regions.^51^ To avoid bias related to the exclusion of striatal/subcortical binding, possibly leading to systematic underestimations in DIAD, we then tried more inclusive target masks considering the cortical regions described above plus (i) basal ganglia and (ii) basal ganglia, occipital lobe and thalamus.

#### Voxelwise group-level comparisons

Voxelwise analyses were performed with SPM12 to compare PIB-PET and FDG-PET binding across groups. The statistical threshold was set *a priori* to be *P* < 0.001 uncorrected for multiple comparisons. Family-wise error correction to *P* < 0.05 at the cluster level was used as an additional thresholding approach. Thresholded spm-T images were converted to Cohen’s *d* effect sizes images with the Computational Anatomy Toolbox (CAT12) for SPM12 (http://www.neuro.uni-jena.de/cat/). Symptomatic participants (both sEOAD and DIAD) were compared to CN correcting for age and sex. When comparing sEOAD versus DIAD participants, multiple combinations of additional covariates were included to test their relative impact on the model, including MMSE, APOE ε4 status and respective global cortical biomarker binding.

#### Voxelwise ICAs

To investigate whether sEOAD and DIAD differed with regard to differential expression of binding patterns, we performed a group independent component analysis (ICA), including scans from both groups, separately for PIB-PET and FDG-PET, with the GIFT toolbox (https://trendscenter.org/software/gift/). A number of components were estimated through the minimum description length (i.i.d sampling) implementation in the toolbox, resulting in *N* = 6 estimated components for FDG-PET and *N* = 12 components for PIB-PET. Analysis was run using the Infomax method to define components and ICASSO with *N* = 10 runs. Both bootstrapping and random initiation were used to select a stable run. All the other settings and parameters were set to defaults. To avoid spatially biased results, images with a restricted field of view were excluded from this analysis, and all images were masked to only keep grey/white matter tissue prior to running the analysis. Resulting component maps were visually inspected and selected if they appeared not artefactual (e.g. ring effects).^55^ Loading coefficients for each subject and component were then compared across sEOAD and DIAD groups with and without the same set of covariates mentioned above to test differences.

### Statistical analysis

Group comparisons of continuous variables between sEOAD, DIAD and CN were tested by ANOVA followed by pairwise Tukey honestly significant difference *post hoc* comparisons. Differences in frequencies for qualitative variables were tested with a chi-squared (*χ*^2^) test. Effect sizes for sEOAD versus DIAD comparisons were calculated with Cohen’s *d* for continuous variables and Cramer’s *V* for discrete variables. All *P*-values were corrected for multiple comparisons with a false discovery rate (FDR) correction. Adjusted group comparisons between loading parameters estimated in the ICA were run using a general linear model with all possible combinations of five covariates of nuisance, including age, sex, MMSE, APOE ε4 status and respective global cortical binding (replicating the voxelwise approach). Partial *η*^2^ and respective *P*-values were generated for each of these analyses relative to the group factor. Plotting of voxelwise analysis was done via MRIcroGL software (https://www.nitrc.org/projects/mricrogl). Colour scales were chosen to be perceptually stable. The remaining plotting was performed in R (v4.0.2) with ggplot2.^56^

#### Sensitivity analyses

We ran two different sets of sensitivity analyses. First, considering some evidence for higher cerebellar PIB-PET binding in DIAD, all the global binding and voxelwise group-level comparisons were repeated using pons as the reference region. Second, the original study inclusion criteria required only sEOAD to be amyloid positive, with no specific amyloid status requirement for DIAN participants. Given that this choice could have biased the PIB-PET comparisons, we repeated the global binding analyses after excluding *N* = 2 amyloid-positive CN and *N* = 9 amyloid-negative DIAD participants, as defined by the A ± status provided by the DIAN PET Core (approach described in Su *et al*.^57^). Additionally, DIAN participants lacking an available DIAN PETCore A ± status at the study time point were also excluded (*N* = 7, *N* = 1 CN and *N* = 6 symptomatic DIAD). This sensitivity analysis was thus performed on a sample including *N* = 99 amyloid-negative CN, *N* = 74 amyloid-positive symptomatic DIAD and *N* = 134 amyloid-positive sEOAD participants.

## Results

sEOAD participants were on average 14 years older at PIB-PET (*d* = 1.91, *P*_FDR_ < 0.001) and 13 years older at symptom onset (*d* = 1.96, *P*_FDR_ < 0.001) than DIAD participants. sEOAD participants had on average 3 more years of education (*d* = 1, *P*_FDR_ < 0.001) than DIAD participants and were more likely to be APOE ε4 carriers (55% versus 28%, *V* = 0.26, *P*_FDR_ < 0.001). The distribution of global CDR total scores at PET was significantly different between groups, with the DIAD group having relatively more moderate/severe cases (*V* = 0.37, *P*_FDR_ < 0.001), and CDR sum of boxes was higher in DIAD (*d* = 0.32, *P*_FDR_ = 0.06). sEOAD and DIAD groups did not differ in sex (*V* = 0.03, *P*_FDR_ = 0.8), symptom duration (*d* = 0.26, *P*_FDR_ = 0.09), CDR sum of boxes scores and MMSE (*d* = 0.33, *P*_FDR_ = 0.06; *d* = 0.08, *P*_FDR_ = 0.6, respectively). See Table 1 for details.

### Global binding group-level comparisons

**Figure 1:**
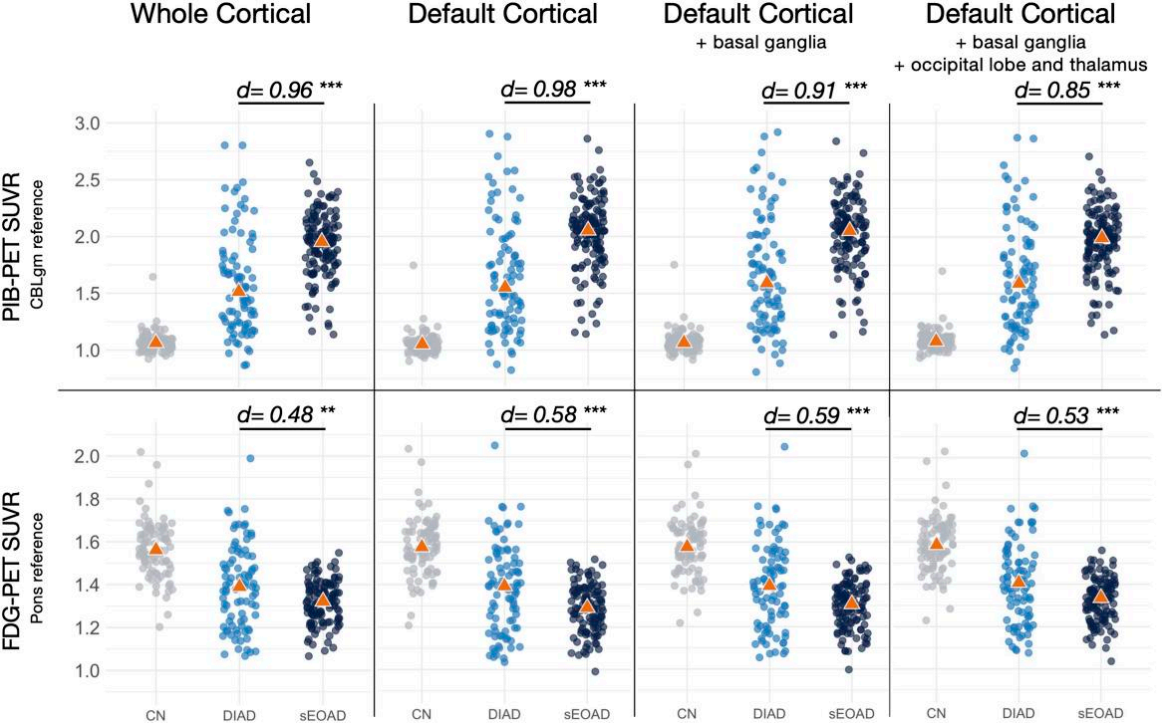
**Global binding group-level comparisons by target region.** Legend: Figure showing distribution of PIB-PET SUVR and FDG-PET SUVR values according to group, with four different target regions (see text for details). See text for more details. Each point represents a participant. CBLgm, cerebellar grey matter; *d*, Cohen’s *d*; ***P < 0.001; **P < 0.01; CN, cognitively normal; DIAD, dominantly inherited Alzheimer’s disease (symptomatic participants); sEOAD, sporadic early-onset Alzheimer’s disease; FDG, ^18^F-fluorodeoxyglucose; PIB, Pittsburgh Compound B; SUVR, standardized uptake value ratio.

sEOAD participants had on average higher whole cortical PIB-PET (*d* = 0.96, *P*_FDR_ < 0.001; see Fig. 1) and lower whole cortical FDG-PET (*d* = 0.48; *P*_FDR_ = 0.002) binding compared with DIAD. Converted Centiloid values from the whole cortical PIB-PET SUVR, with cerebellar reference, were mean ± SD 94 ± 32 in sEOAD and 55 ± 50 in DIAD (see the Supplementary materials for Centiloid conversion validation). PIB-PET differences remained significant after adjusting for various combinations of age, sex, MMSE and APOE ε4 status covariates. FDG-PET differences remained significant when correcting for sex but not in the other models (see Supplementary Table 1). PIB-PET differences also remained significant with different target regions, ranging from the greatest magnitude using a default cortical target with cerebellar reference (*d* = 0.98, *P*_FDR_ < 0.001) to the smallest magnitude using the most comprehensive target region including default regions plus basal ganglia, occipital lobe and thalamus (*d* = 0.85, *P*_FDR_ < 0.001); see Fig. 1 for details. Within DIAD participants, global binding estimations varied between and within mutated genes (see Supplementary Fig. 1). Several DIAD participants presented with a seemingly negative amyloid-PET scan (see Fig. 1 and ‘Sensitivity analysis’ section below). In a subset analysis, selecting only the PSEN-1 variants with mutations occurring prior to codon < 200, the differences for whole cortical PIB-PET biding were not significant compared with the sEOAD (*P* = 0.17) (see Supplementary Fig. 1).

### Voxelwise group-level comparisons

**Figure 2:**
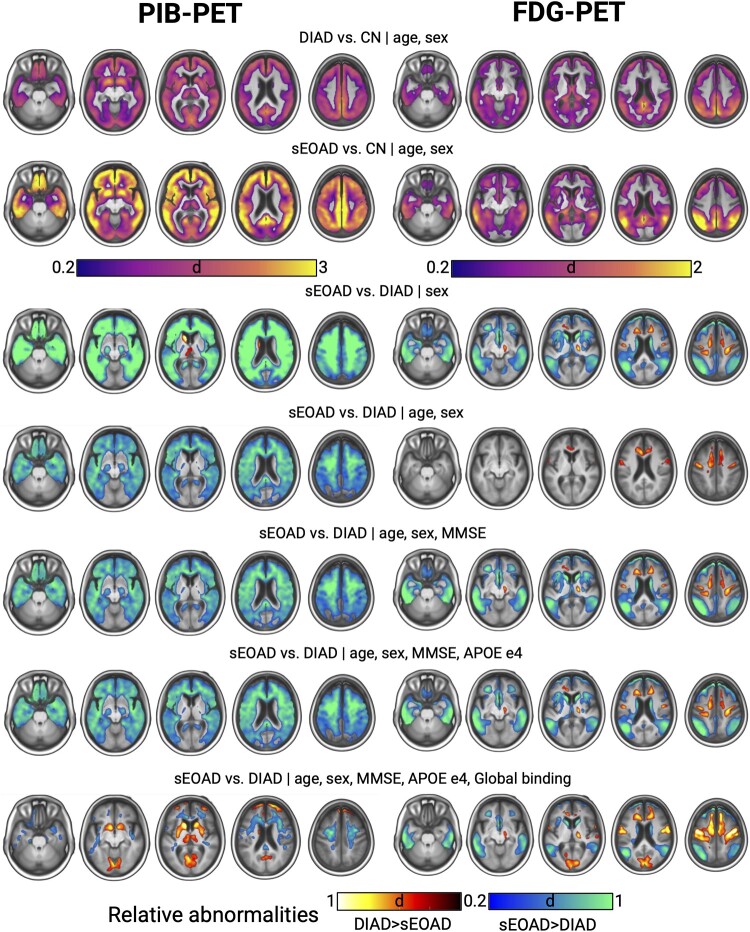
**Voxelwise group-level comparisons with different sets of covariates.** Legend: Figure showing voxelwise group comparisons results between sEOAD/DIAD and CN groups (top two rows) and between sEOAD and DIAD participants (bottom five rows). For the DIAD versus sEOAD comparisons, models adjusted with different sets of covariates are shown. Colour scales represent Cohen’s *d* effect sizes; see main text for details. Images are generated with mricroGL software. *d*, Cohen’s *d*; CN, cognitively normal; DIAD, dominantly inherited Alzheimer’s disease; sEOAD, sporadic early-onset Alzheimer’s disease; MMSE, Mini-Mental State Examination; APOE, apolipoprotein E; FDG, ^18^F-fluorodeoxyglucose; PIB, Pittsburgh Compound B.

Both sEOAD and DIAD groups showed elevated PIB-PET and reduced FDG-PET compared with the CN group, with sEOAD overall showing greater effect sizes but similar spatial patterns (Fig. 2). When comparing sEOAD versus DIAD directly, the fully adjusted models showed significant tracer-specific regional differences. For PIB-PET, DIAD showed relatively greater binding in occipital, frontal and subcortical regions, including caudate, putamen and thalamus, whereas sEOAD participants showed relatively greater binding restricted to WM. For FDG-PET, sEOAD participants showed greater hypometabolism in Alzheimer’s disease signature regions and the caudate nuclei, with DIAD instead showing greater hypometabolism in precentral, medial frontal areas and occipital regions. These observed differences showed some variation with the inclusion of different combinations of covariates (see Fig. 2).

### Voxelwise ICA

**Figure 3:**
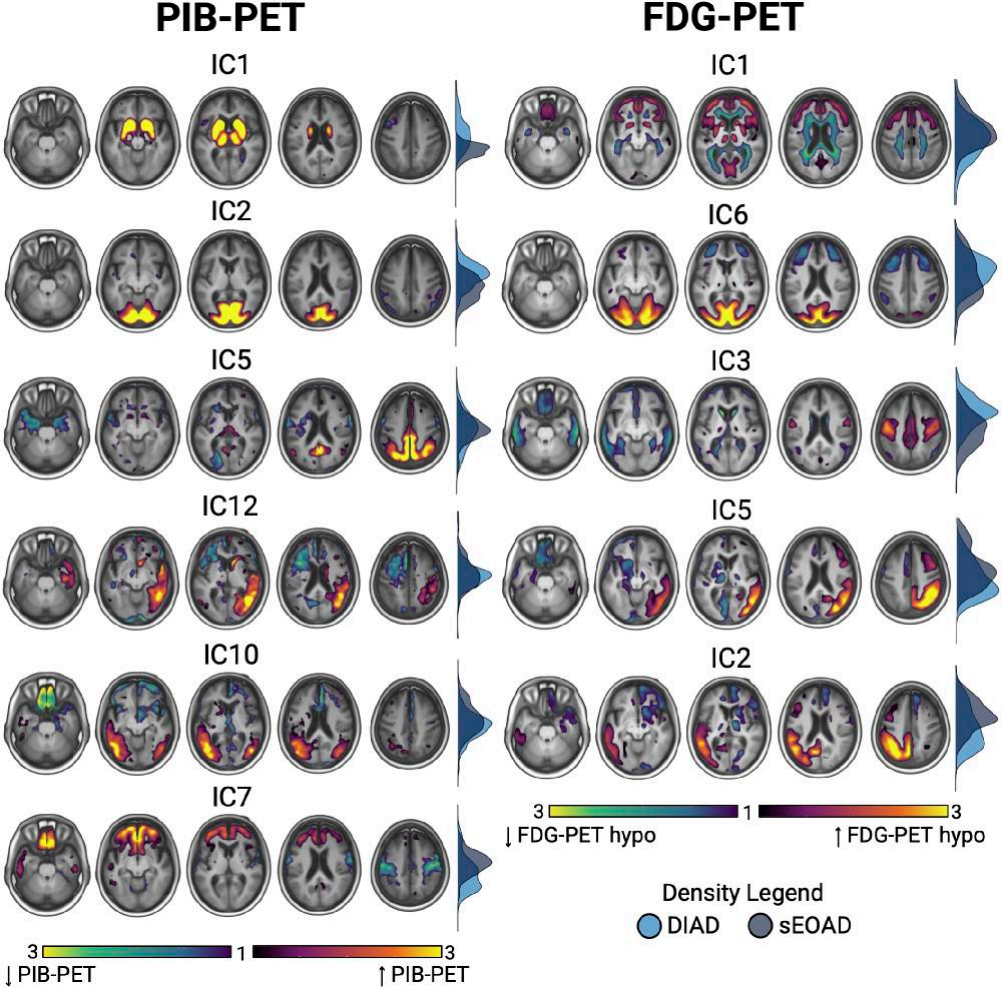
**Voxelwise ICA. Legend: Figure showing results of the ICAs.** Brain renderings show components for both PIB-PET (left) and FDG-PET (right), with colour scales representing *z*-scored contributions of each voxel to the respective component. Components were thresholded to |*z*| > 1 for visualization. Density plots for each component represent distribution of loading parameters between DIAD and sEOAD participants. Images are generated with mricroGL software. IC, independent component; DIAD, dominantly inherited Alzheimer’s disease; sEOAD, sporadic early-onset Alzheimer’s disease; FDG, ^18^F-fluorodeoxyglucose; PIB, Pittsburgh Compound B.

**Figure 4:**
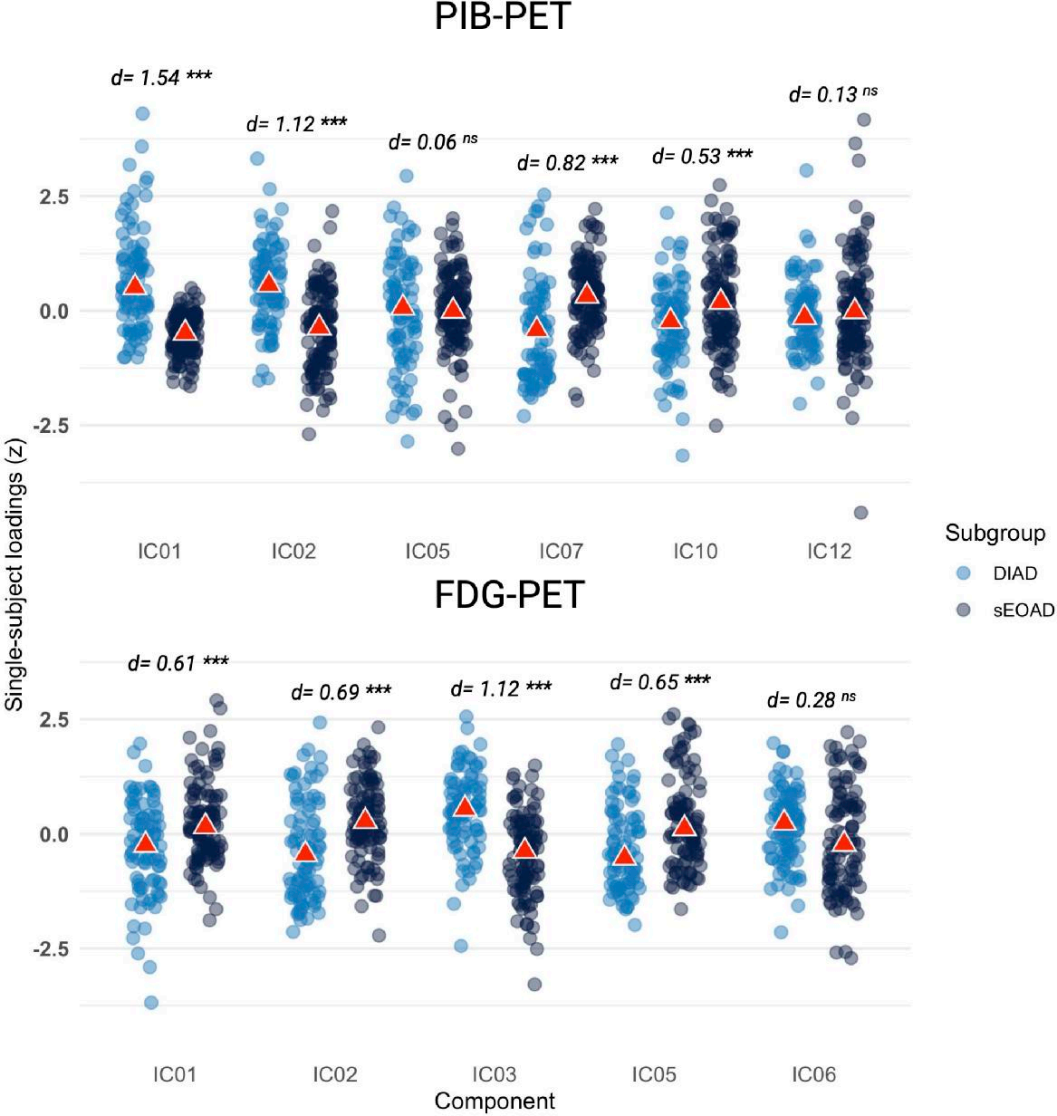
**Distribution of loadings for PIB-PET and FDG-PET components.** Legend: Figure showing distribution of loadings in sEOAD and DIAD participants for all the identified PIB-PET and FDG-PET components. Triangles indicate respective medians. Effect size and significance refer to crude (unadjusted) comparisons. Each point represents a participant. *d*, Cohen’s *d*; ***P_FDR_ < 0.001; ns, non-significant; IC, independent component; DIAD, dominantly inherited Alzheimer’s disease; sEOAD, sporadic early-onset Alzheimer’s disease; FDG, ^18^F-fluorodeoxyglucose; PIB, Pittsburgh Compound B.

Six components for PIB-PET and five components for FDG-PET were selected after visual inspection (see Fig. 3). For PIB-PET, unadjusted comparisons of loading parameters showed that DIAD participants on average expressed more significantly the basal ganglia (IC1, *d* = 1.54, *P*_FDR_ < 0.001) and occipital (IC2, *d* = 1.12, *P*_FDR_ < 0.001) components, whereas sEOAD participants expressed more significantly the frontal (IC7, *d* = 0.82, *P*_FDR_ < 0.001) and left temporoparietal components (IC10, *d* = 0.53, *P*_FDR_ < 0.001). The bilateral superior parietal (IC5) and the right temporoparietal (IC12) components were similarly expressed in the two groups (*d* = 0.06, *P*_FDR_ = 0.7; *d* = 0.13, *P*_FDR_ = 0.4, respectively). For FDG-PET, DIAD participants expressed more significantly the pericentral (IC3, *d* = 1.12, *P*_FDR_ < 0.001) component, whereas sEOAD participants expressed more significantly the frontal (IC1, *d* = 0.60, *P*_FDR_ < 0.001), left temporo-parieto-frontal (IC2, *d* = 0.69, *P*_FDR_ < 0.001) and right temporo-parieto-frontal (IC5, *d* = 0.65, *P*_FDR_ < 0.001) components. The occipital component was expressed similarly in both groups, with a trend towards higher expression in DIAD (*d* = 0.28, *P*_FDR_ = 0.06). See Fig. 4 for the distribution of loadings for both PIB-PET and FDG-PET components. Differences for the basal ganglia and occipital PIB-PET components and for the pericentral and right temporo-parieto-frontal FDG-PET components remained significant after adjusting for various combinations of age, sex, MMSE, *APOE* ε4 status and respective global cortical binding (see Supplementary Table 2). Supplementary Fig. 4 shows in more detail the distribution of scores across the four components significant in adjusted models, labelling DIAD patients showing the highest loadings according to their mutation.

### Sensitivity analyses

PIB-PET differences were attenuated but still significant when repeated using the pons as reference, across the different target regions in the global analysis (Supplementary Fig. 2) as well as in the voxelwise models (Supplementary Fig. 3). sEOAD participants still showed higher global PIB-PET binding compared with symptomatic amyloid-positive DIAD patients with a greater magnitude of difference using cerebellum versus pons reference regions. As in the main analysis, adding basal ganglia, occipital and thalamic regions to the global PIB region of interest attenuated group differences, as did the use of the pons as the reference region (Cohen’s *d* range 0.86–0.24, *P*_FDR_ range < 0.001–0.15; see Supplementary Fig. 5).

## Discussion

sEOAD and DIAD patients showed overlap and also significant differences in both PIB-PET and FDG-PET binding patterns. PiB-PET retention, a measure of Aβ plaque accumulation, largely converged in the same regions for both groups, with particularly pronounced involvement of subcortical structures in dominantly inherited cases. Brain glucose hypometabolism in pericentral and medial frontal regions was relatively more pronounced in dominantly inherited rather than sporadic participants, whereas sEOAD showed relatively greater temporoparietal and caudate involvement. The present findings suggest that the presence or absence of known dominantly inherited mutations may influence Aβ plaque accumulation and brain glucose hypometabolism patterns in EOAD. Alternatively, Aβ plaque characteristics (e.g. cotton wool plaques) may differ according to DIAD variant, which may explain heterogeneity in PIB affinity and lower PIB-PET binding affinity in certain DIAD variants.

Previous studies have focused on differences between DIAD and LOAD, in which differences in age may be a significant confounder convolving age-related processes with possible differences due to mutation status. The present study was designed to compare DIAD and sEOAD cohorts sharing a younger age at symptom onset, thereby disentangling differences more likely to be related to dominantly inherited disease. Taking into account that DIAD participants were still significantly younger than the sEOAD participants, we found overall higher global PIB-PET binding in sEOAD compared with DIAD. The magnitude of this difference was influenced by the choice of target and reference regions but was overall consistent across methods and in line with previous reports.^26,58-63^ The observed PIB-PET binding differences may also possibly reflect heterogeneity in relationships between symptom onset and amyloid duration/time, the latter potentially longer in sEOAD compared with DIAD.^64,65^ In assessing differences in the spatial topography of amyloid deposition, voxelwise analyses (controlling for global PIB-PET binding, thus revealing regions relatively more involved) demonstrated that DIAD showed greater involvement of striatum and thalamus, as well as the occipital pericalcarine region and the frontal pole, with sEOAD patients showing higher white matter PIB-PET binding. The striatum is known to be involved early in DIAD,^58,62,66-71^ even before detectable cortical accumulation,^58,62,66-68^ with the thalamus also recognized as a key structure in the progression of DIAD.^19,59,71^ This is in contrast with the relatively late detection of significant amyloid-PET binding in the striatum in patients with sporadic Alzheimer’s disease.^67,72,73^ Similar findings were previously reported *in vivo* with PIB-PET in a smaller cohort including a mix of sporadic LOAD and EOAD and DIAD *PSEN1* mutation carriers,^59^ with structural MRI providing converging evidence for greater thalamic volume loss in DIAD versus sEOAD in an independent study.^74^ Concordant evidence was also reported in a previous autopsy study, which examined sporadic LOAD and a mix of *APP* and *PSEN1* DIAD patients, finding more severe striatal Aβ42 accumulation in DIAD and more severe cortical Aβ42 accumulation in sporadic Alzheimer’s disease.^75^ Although mutation carriers with the APP E693Q variant (Dutch-type CAA) were not included in this analysis, greater PIB binding in occipital regions in DIAD could be related to underlying cerebral amyloid angiopathy,^76,77^ which shows a predilection for occipital cortex and can be more severe in DIAD compared with sEOAD.^78^ In particular, *PSEN1* mutations occurring after codon 200 more frequently associate with amyloid angiopathy and parenchymal amyloid accumulation including both diffuse and cored plaques.^79^ It is difficult to interpret the relatively higher white matter PIB-PET binding observed in sEOAD compared with DIAD. It has been proposed that amyloid-PET ligand binding in white matter may be associated with myelin or myelin-associated proteins, with lower binding seen in regions that show T2/FLAIR white matter hyperintensities on MRI.^80-82^ Our observation could thus indicate greater white matter pathology in the DIAD group,^83,84^ although further work is needed to understand the biological substrate of the detected differences in white matter binding. Overall, our findings suggest that, within the Alzheimer’s disease spectrum, genetic determinants influence the topography and magnitude of PIB-PET binding patterns.

Differences in the degree and spatial patterns of PIB-PET binding between DIAD and sEOAD may in part be related to mutation-specific differences in Aβ fibril microstructure and available binding sites^26,85,86^ rather than differences in the burden or distribution of Aβ pathology *per se*. Specific DIAD mutations lead to ‘cotton wool’ amyloid plaques, which show an absent-to-low affinity for PIB binding.^85^ Additionally, PIB-PET binding in Alzheimer’s disease signature regions may be reflecting different amyloid pathology in DIAD versus sporadic Alzheimer’s disease, namely diffuse plaques in the former and a combination of diffuse and cored plaques in the latter.^26^ It is also possible that different brain regions may have greater susceptibility to the mechanisms posited to trigger Aβ plaque accumulation, i.e. overproduction of Aβ peptides in DIAD versus reduced clearance of Aβ in sporadic Alzheimer’s disease.^87-90^ In keeping with this hypothesis, a previous study has found that regional Aβ42 levels at autopsy correlated with synaptic markers in sporadic Alzheimer’s disease but with the APP/β-C-terminal fragment of APP in DIAD.^75^

Within the DIAD group, we found further evidence of how different mutations lead to different patterns of Aβ plaque accumulation.^91^ We observed heterogeneity in PIB-PET binding patterns, implying that not only mutation status but also mutation position (e.g. within the *PSEN1* gene) may influence regional vulnerability to Aβ pathology. This confirms previous observations^59,62,91^ and could indicate that different mutations may generate downstream Aβ pathology with distinct conformations, biochemical properties and regional distributions. Amyloid pathology heterogeneity in DIAD has also been reported in autopsy studies,^78,79,92,93^ demonstrating that *PSEN1* individuals with mutations occurring after codon 200 show more cored plaques and more amyloid angiopathy and that individuals with missense *APP* mutations show greater plaque formation than individuals with *APP* duplication, with *APP* individuals in general showing more severe Aβ40 angiopathy. Finally, a previous in vitro study has demonstrated that the Aβ42 and Aβ40 production potential differed across various mutant *PSEN1* proteins.^94,95^ Previous studies have also highlighted how, even within the same families, the same mutation may present with different PET binding patterns, pathology and clinical profiles.^96-98^ Finally, in our population, a few symptomatic DIAD mutation carriers presented with a seemingly negative amyloid-PET scan. This has been described previously^63,99-101^ and may be associated with an Aβ pathology type being under-detected by PIB-PET (e.g. cotton wool plaques), amyloid accumulation in the cerebellar/pontine reference regions leading to low SUVR values^26^ or symptoms in DIAD individuals that are not directly related to Aβ pathology.^26,85^ Overall, our data demonstrate that the ability to quantify amyloid burden with PIB-PET likely varies according to the specific DIAD mutation and may explain observed differences within DIAD variants (codon <200 versus >200) and in relation to sEOAD. Previous studies in the DIAN cohort suggest that PIB-PET has limited ability to detect Aβ aggregates in cotton wool plaques and may underestimate the total Aβ plaque burden in brain regions with abundant cotton wool plaques. *PSEN1* mutations occurring after codon 200 have been reported to have a higher frequency of cotton wool plaques relative to those occurring before codon 200, and our study shows that *PSEN1* > 200 codon mutations also tend to present overall lower PIB-PET SUVR.

Adjusting for global cortical glucose metabolism, DIAD participants showed greater hypometabolism in the medial occipital, thalamic and pericentral cortex. sEOAD participants showed greater hypometabolism in Alzheimer’s disease signature temporoparietal regions as well as the caudate nucleus, consistent with previously described greater involvement of subcortical structures in sEOAD compared with sporadic LOAD.^102^ The discordance between significant Aβ pathology and lack of concomitant neurodegeneration in some subcortical structures (e.g. the caudate nuclei) in DIAD is consistent with previous observations.^58,66,103^ Greater pericentral hypometabolism in DIAD could be associated with motor cortex and spinal cord involvement in some DIAD mutations, manifesting clinically as spasticity, myelopathy or myoclonus.^10-12^ Conversely, these structures are largely spared in sporadic Alzheimer’s disease.^104-107^ Interestingly, the DIAD participant expressing the highest loading for the pericentral FDG-PET component carried a mutation in *PSEN1* (Ser169Leu) that has been associated with clumsiness and myoclonus.^108^ Our analyses also indicated that sEOAD patients more frequently expressed a right-dominant pattern of temporoparietal hypometabolism compared with DIAD patients. This is likely to be associated with a higher prevalence of atypical (i.e. non-amnestic) and asymmetric Alzheimer’s disease clinical presentations in sEOAD compared with DIAD,^3,47^ which are tightly associated with specific FDG-PET hypometabolism patterns.^34,109-111^

This study has several limitations. First, it remains to be fully elucidated whether observed differences in amyloid-PET patterns may reflect absolute differences in either presence, location or type of Aβ pathology. Autopsy studies have generally indicated more severe pathology in DIAD versus all age sporadic Alzheimer’s disease.^21,92,96,112,113^ Additionally, previous fluid biomarker studies have provided evidence for lower^114^ or similar^13,47^ CSF Aβ_42_ in DIAD versus sporadic Alzheimer’s disease, although CSF Aβ37, Aβ38 and Aβ39 concentrations were reported to be lower in DIAD.^13^ It is possible that the inconsistency between imaging-, fluid- and autopsy-based findings is associated with both operational factors (e.g. analytical pipelines, reference and target regions), as well as biological factors (e.g. differential sensitivity of amyloid-PET tracers to different Aβ aggregates, type of mutation and disease stage).^26,85,86^ We tried to address these factors by considering different target and reference regions, as well as by running pattern-based analyses such as ICA, which overall provided convergent evidence. Second, this study likely suffers from sampling bias. sEOAD is frequently diagnosed years after symptom onset, with a sometimes unreliable estimation of the actual duration from symptom onset. Conversely, DIAD participants, especially in studies like DIAN, are prospectively followed before and after symptom onset and closely monitored, implying a very accurate estimation of symptom duration. Therefore, despite the similar duration of estimated symptoms from onset in sEOAD and DIAD groups in the present study, it is still possible that due to a recall bias, the sEOAD participants had a longer duration of symptomatic disease at the time of imaging, potentially contributing to some of the observed PIB-PET and FDG-PET differences. Notwithstanding this uncertainty, our analyses (i) incorporated chronological age at PIB-PET, thus using a reliably anchored covariate, and (ii) focused on relative pattern differences, which were by design unrelated to global PET binding. Third, CN participants included in the present study were asymptomatic, non-mutation carriers from DIAN and were significantly younger than our sEOAD patients. All our primary analyses, however, only involved direct comparisons between symptomatic participants. Fourth, the symptomatic DIAD group presented with intrinsic heterogeneity with regard to PIB-PET binding patterns, potentially associated with the given mutation. It is reasonable to expect that some DIAD mutations may present with overall higher PIB-PET binding compared to sEOAD, but larger sample sizes will be needed in order to systematically investigate this.

## Conclusion

In conclusion, our findings indicate that sporadic and dominantly inherited EOAD show overlap and also important distinctions in patterns of Aβ plaque deposition and glucose hypometabolism. Amyloid-PET differences likely reflect the interplay between heterogeneity in the biochemical and microstructural properties of Aβ deposits, regional vulnerabilities and PET tracer binding properties. Future studies including additional biomarkers (e.g. tau- and neuroinflammation-PET) may provide further insights into common and distinct aspects of the Alzheimer’s disease pathophysiological cascade in sporadic versus dominantly inherited disease.

## Supplementary Material

fcae159_Supplementary_Data

## Data Availability

Data supporting the findings of this study are available on request and will follow the policies of the DIAN (https://dian.wustl.edu) and of the UCSF ADRC (https://memory.ucsf.edu/research-trials/professional/open-science#Data-Sharing), both of which comply with the guidelines established by the Collaboration for Alzheimer’s Prevention. Data are not publicly available in order to preserve the privacy of research participants.
