# Supplementary material for: Molecular neuroimaging in dominantly inherited versus sporadic early-onset Alzheimer’s disease
Source: Brain Commun. 2024 May 3;6(3):fcae159. doi: 10.1093/braincomms/fcae159 (PMC11114609; doi:10.1093/braincomms/fcae159)
Supplement: fcae159_Supplementary_Data [file fcae159_supplementary_data.docx]

# Supplementary material.

**Molecular Neuroimaging in Dominantly Inherited versus Sporadic Early-Onset AD**

Leonardo Iaccarino^1*^, Jorge J Llibre-Guerra^2,3*^, Eric McDade^2,3^, Lauren Edwards^1^, Brian Gordon^4^, Tammie Benzinger^4^, Jason Hassenstab^2,3^, Joel H. Kramer^1^, Yan Li^5^, Bruce L. Miller^1^, Zachary Miller^1^, John C. Morris^2,3^, Nidhi Mundada^1^, Richard J. Perrin^6^, Howard J. Rosen^1^, David Soleimani-Meigooni^1^, Amelia Strom^1^, Elena Tsoy^1^, Guoqiao Wang^5^, Chengjie Xiong^5^, Ricardo Allegri^7^, Patricio Chrem^7^, Silvia Vazquez^7^, Sarah B. Berman^8^, Jasmeer Chhatwal^9^, Colin L Masters^10^, Martin R. Farlow^11^, Mathias Jucker^12^, Johannes Levin^13,14,15^, Stephen Salloway^16^, Nick C. Fox^17^, Gregory Day^18^, Maria-Luisa Gorno-Tempini^1^, Adam L. Boxer^1^, Renaud La Joie^1^ , Randall Bateman^2,3*^ ,Gil D. Rabinovici^1,19*^.

**^*^ These authors have contributed equally**

**Supplementary Table of Content**

- **Supplementary Info 1**. Centiloid validation for Global Cortical PIB-PET SUVR
- **Supplementary Table 1**. PIB-PET and FDG-PET Global Cortical comparisons with different sets of covariates
- **Supplementary Table 2**. PIB-PET and FDG-PET ICA components loadings comparisons with different sets of covariates
- **Supplementary Figure 1**. Global PIB-PET estimations across sEOAD and DIAD participants according to mutation
- **Supplementary Figure 2**. Global PIB-PET binding group-level comparisons by reference regions
- **Supplementary Figure 3**. PIB-PET voxelwise group-level comparisons with different sets of covariates and reference regions
- **Supplementary Figure 4**. Labeled distribution of loadings in significant PIB-PET and FDG-PET components
- **Supplementary Figure 5**. Global binding group-level comparisons by target and reference regions in subset of participants

**Supplementary Info 1**. Centiloid validation for Global Cortical PIB-PET SUVR


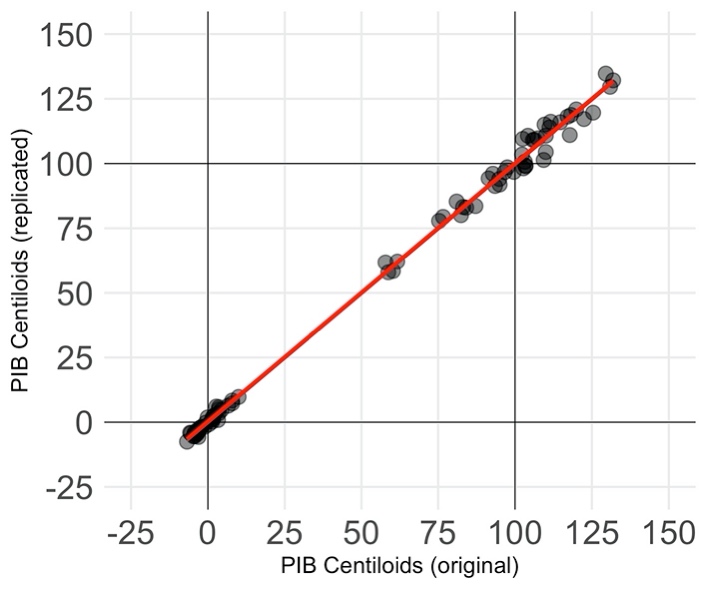


*Level 1.* We first replicated Level-1 analysis following instructions in ^115^, however using SPM12 and not SPM8 for the image processing. We downloaded the 79 PiB-PET 50-70 min scans from GAAIN, estimating neocortical SUVRs using the whole cerebellum reference region. The individual absolute differences with GAAIN data ranged between 0.01%<x<3.93%, meeting the requirement (<5%). We observed a mean PIBwc SUVR for the AD participants of 2.078 (original 2.076) and for the YC of 1.011 (original 1.009).

Linear fit for observed and published GAAIN Centiloids

Based on this, our PIB Centiloid conversion formula was:

$$PIBCL=100*\left( PIBSUVRind-1.011 \right)/1.067$$

We then estimated Centiloids, yielding a linear fit with the published Centiloid values meeting all the requirements ^115^, i.e., an R^2^ of 0.997 (required >.98), a slope of 0.998 (required between 0.98<x<1.02) and an intercept of 0.11 (required between -2<x<2) (see also Figure 1).

*Level 2.* To calibrate the Centiloid conversion formula, we processed the 79 GAAIN PIB-PET scans as described in the main text, excluding one case from subsequent steps due to Freesurfer 7.1 parcellation failure. PIB-PET standard and local Global Cortical SUVRs were highly correlated (R^2^:0.9804, required >0.7). Based on slope and intercept, we converted local PIB-PET SUVRs in standard PIB-PET SUVRs (Eq 2.2.3.2b), then applied the Centiloid conversion formula (Eq 2.2.3).

$$Eq. 2.2.3.2b: PIBCalcSUVRIND=\frac{\left( PIBLocalSUVRind -0.25893 \right)}{0.82857}$$

$$Eq. 2.2.3: PIBCalcCL=100*\frac{\left( PIBCalcSUVRIND-1.011 \right)}{1.067}$$

Based on the converted Centiloids, the final formula to directly convert local Global Cortical PIB-PET SUVRs to Centiloids was (see also Figure below):

$$PIBCL=\left( 113.1*PIBGlobCorticalSUVR \right)-124$$

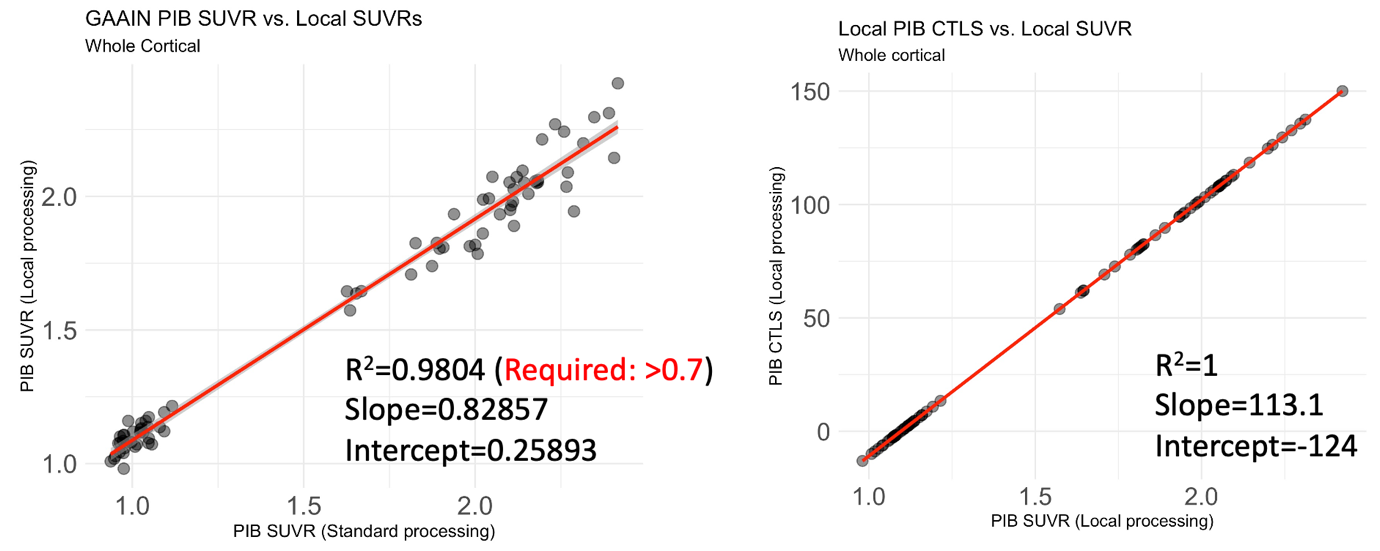


PIB-PET local global cortical SUVR validation

Supplementary Table 1. PIB-PET and FDG-PET Global Cortical comparisons with different sets of covariates

| Variable | Mod | Covariates | *η*p ² | Beta | SE | T | P |
| --- | --- | --- | --- | --- | --- | --- | --- |
| Whole Cortical binding | PIB | sex | 0.19 | 0.34 | 0.05 | 7.09 | <0.001 |
|  |  | age/sex | 0.09 | 0.32 | 0.07 | 4.73 | <0.001 |
|  |  | age/sex/MMSE | 0.10 | 0.33 | 0.07 | 5.03 | <0.001 |
|  |  | age/sex/MMSE /APOE e4 status | 0.11 | 0.33 | 0.07 | 4.99 | <0.001 |
|  | FDG | sex | 0.05 | -0.07 | 0.02 | -3.39 | 0.001 |
|  |  | age/sex | 0.001 | 0.01 | 0.03 | 0.49 | 0.6 |
|  |  | age/sex/MMSE | 0.000008 | 0.00 | 0.02 | 0.04 | 1.0 |
|  |  | age/sex/MMSE /APOE e4 status | 0.0001 | 0.00 | 0.02 | 0.14 | 0.9 |

Table showing results of group comparisons of global cortical PIB-PET and FDG-PET SUVR with different sets of covariates, replicating the voxelwise analyses.

Legend: IC=Independent Component; PIB=PIB-PET; FDG=FDG-PET; MMSE=Mini-Mental State Examination; APOE= Apolipoprotein E; *η*p ²=Partial eta^2^

Supplementary Table 2. PIB-PET and FDG-PET ICA components loadings comparisons with different sets of covariates

| Component | Mod | Covariates | *η*p ² | Beta | SE | T | P |
| --- | --- | --- | --- | --- | --- | --- | --- |
| IC01 | PIB | sex | 0.37 | -1.23 | 0.11 | -11.01 | <0.001 |
|  |  | age/sex | 0.16 | -0.92 | 0.15 | -6.15 | <0.001 |
|  |  | age/sex/MMSE | 0.15 | -0.90 | 0.15 | -6.01 | <0.001 |
|  |  | age/sex/MMSE/APOE e4 status | 0.16 | -0.91 | 0.15 | -6.03 | <0.001 |
|  |  | age/sex/MMSE/APOE e4 status/Global Cortical PIB-PET SUVR | 0.13 | -0.88 | 0.16 | -5.43 | <0.001 |
| IC02 |  | sex | 0.24 | -0.98 | 0.12 | -7.95 | <0.001 |
|  |  | age/sex | 0.12 | -0.90 | 0.17 | -5.31 | <0.001 |
|  |  | age/sex/MMSE | 0.12 | -0.89 | 0.17 | -5.31 | <0.001 |
|  |  | age/sex/MMSE/APOE e4 status | 0.12 | -0.88 | 0.17 | -5.15 | <0.001 |
|  |  | age/sex/MMSE/APOE e4 status/Global Cortical PIB-PET SUVR | 0.08 | -0.75 | 0.18 | -4.20 | <0.001 |
| IC03 | FDG | sex | 0.25 | -0.97 | 0.12 | -7.83 | <0.001 |
|  |  | age/sex | 0.07 | -0.61 | 0.16 | -3.70 | <0.001 |
|  |  | age/sex/MMSE | 0.08 | -0.63 | 0.16 | -3.92 | <0.001 |
|  |  | age/sex/MMSE/APOE e4 status | 0.08 | -0.65 | 0.16 | -4.05 | <0.001 |
|  |  | age/sex/MMSE/APOE e4 status/Global Cortical FDG-PET SUVR | 0.08 | -0.65 | 0.16 | -4.08 | <0.001 |
| IC05 |  | sex | 0.10 | 0.62 | 0.14 | 4.46 | <0.001 |
|  |  | age/sex | 0.03 | 0.46 | 0.19 | 2.46 | 0.01 |
|  |  | age/sex/MMSE | 0.04 | 0.50 | 0.18 | 2.80 | 0.006 |
|  |  | age/sex/MMSE/APOE e4 status | 0.04 | 0.51 | 0.18 | 2.80 | 0.006 |
|  |  | age/sex/MMSE/APOE e4 status/Global Cortical FDG-PET SUVR | 0.06 | 0.51 | 0.16 | 3.28 | 0.001 |

Table showing results of group comparisons of loadings for PIB-PET and FDG-PET components surviving different sets of covariates, replicating the voxelwise analyses.

Legend: IC=Independent Component; PIB=PIB-PET; FDG=FDG-PET; MMSE=Mini-Mental State Examination; APOE= Apolipoprotein E; *η*p ²=Partial eta^2^

Supplementary Figure 1. Global PIB-PET estimations across sEOAD and DIAD participants according to mutation


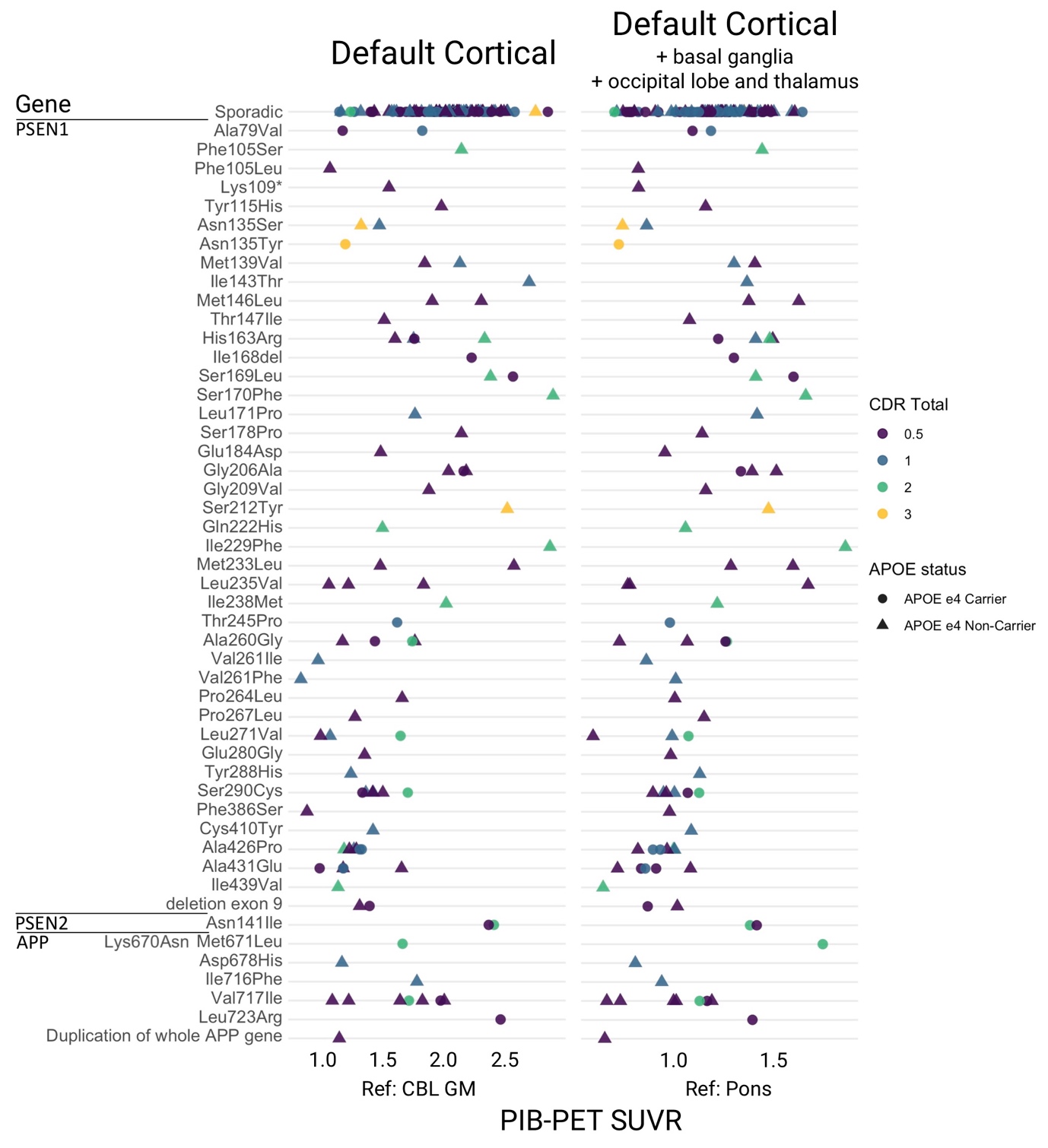


Figure showing estimations of PIB-PET binding with two different approaches, default cortical average with cerebellar gray matter reference (left) and default cortical plus basal ganglia, occipital lobe and thalamus with pons reference (right). These approaches were selected for this plot as they are likely to respectively favor higher SUVRs in sEOAD or DIAD participants. Values for DIAD participants are shown split by mutation and sorted by codon.

Legend: *PSEN1*=Presinilin-1; *PSEN2*=Presinilin-2; *APP*=Amyloid Precursor Protein; CBL GM= Cerebellar Gray Matter; CDR= Clinical Dementia Rating; APOE= Apolipoprotein E

Supplementary Figure 2. Global PIB-PET binding group-level comparisons by reference regions


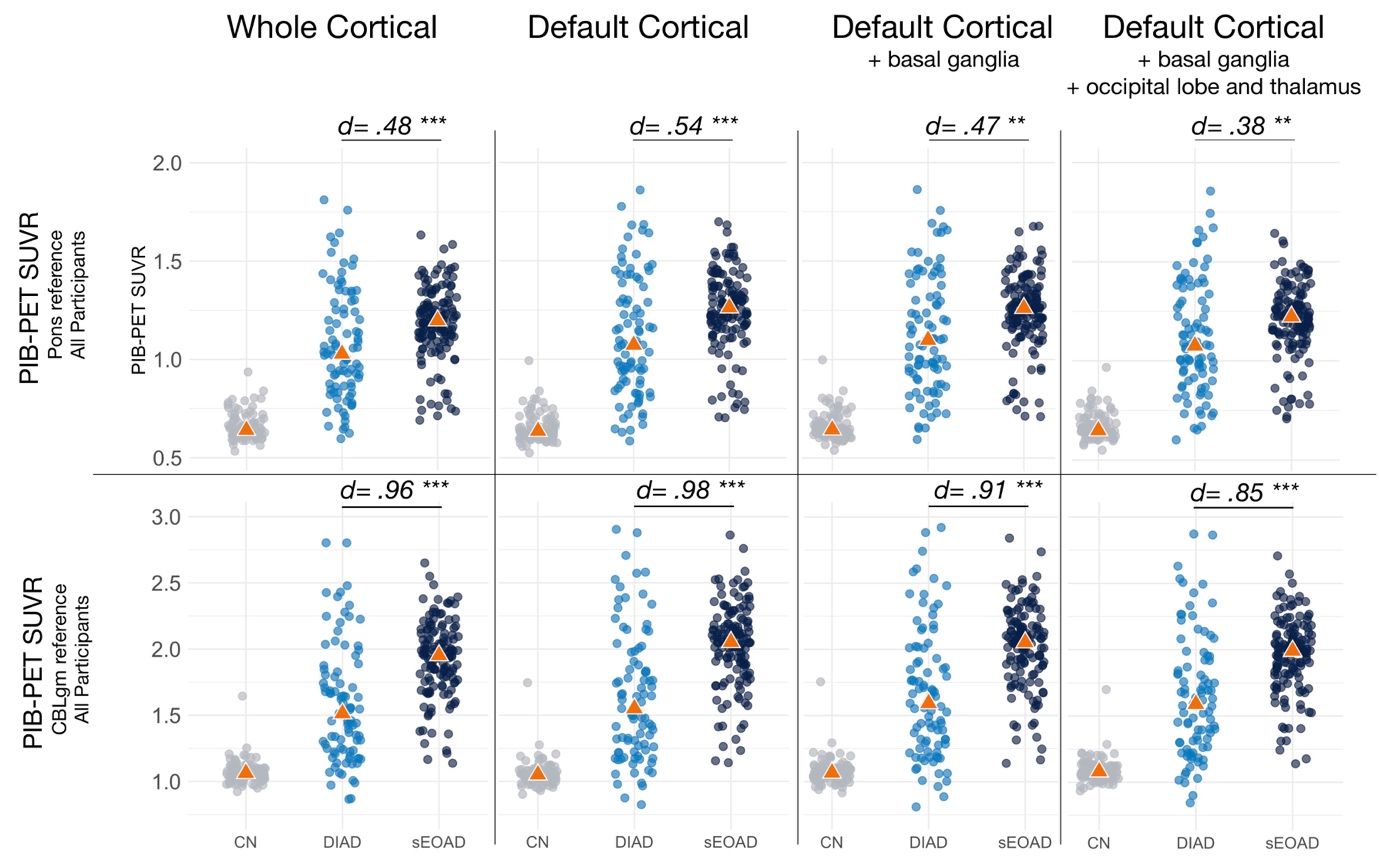


Figure showing distribution of PIB-PET SUVR values according to group, with 8 different combinations of target and reference regions. The default cortical mask used in the study is described in ^52^.

Legend: CBL= Cerebellum; d= Cohen’s d; ***=p<0.001; ** p<0.01; CN=Cognitively Normal; DIAD=Dominantly Inherited Alzheimer’s Disease; sEOAD= Sporadic Early Onset Alzheimer’s Disease

Supplementary Figure 3. PIB-PET voxelwise group-level comparisons with different sets of covariates and reference regions


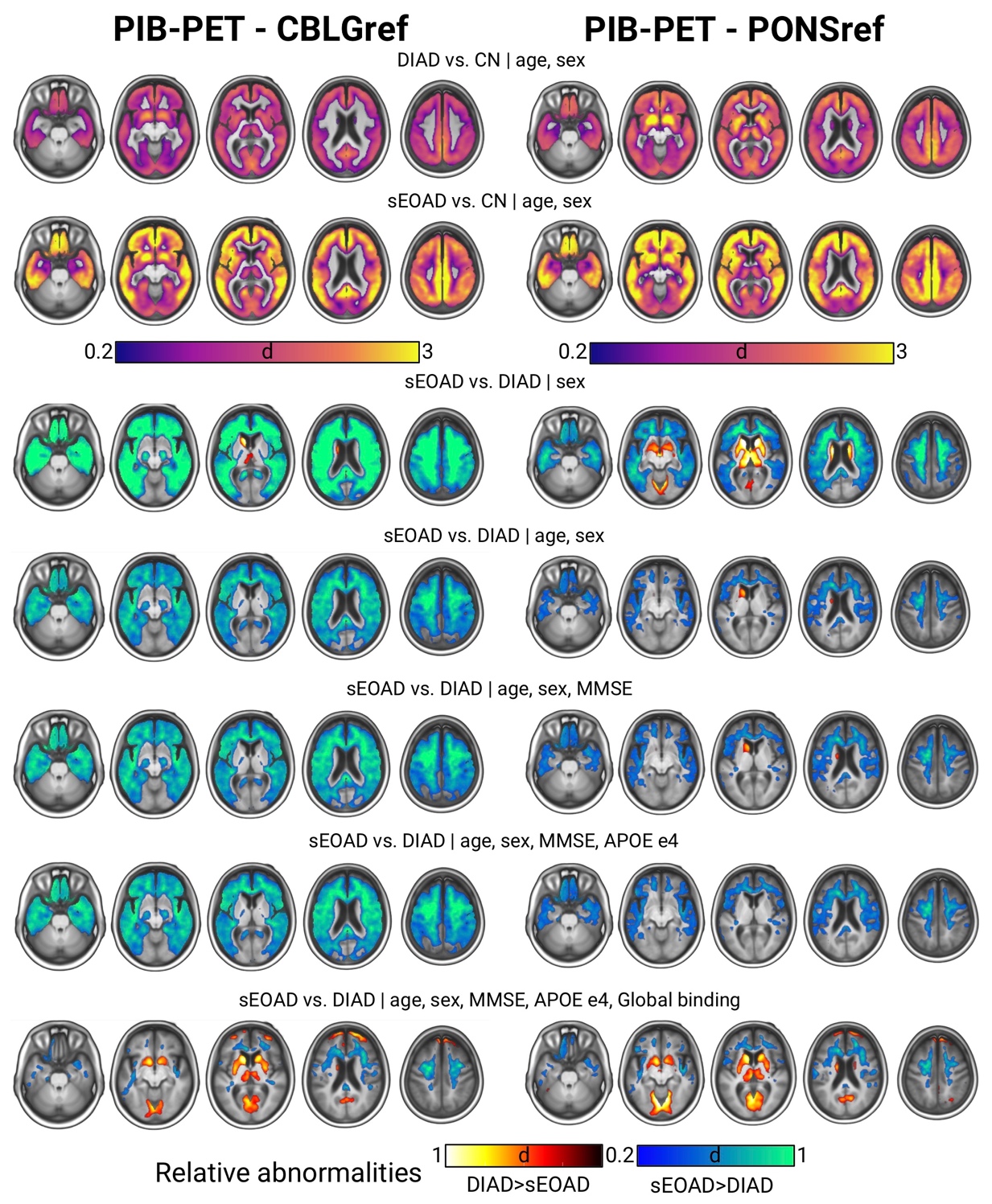


Figure showing PIB-PET voxelwise group comparisons results between sEOAD/DIAD and CN groups (top two rows) and between sEOAD and DIAD participants (bottom five rows). For the DIAD vs. sEOAD comparisons models adjusted with different sets of covariates are shown. Analyses are presented using both cerebellar GM (left) and pons (right) as reference region. Colorscale represent Cohen’s d effect sizes, see main text for details. Images are generated with mricroGL software.

Legend: d= Cohen’s d; CN=Cognitively Normal; DIAD=Dominantly Inherited Alzheimer’s Disease; sEOAD= Sporadic Early Onset Alzheimer’s Disease; MMSE=MiniMental State Examination; APOE= Apolipoprotein E;

Supplementary Figure 4. Labeled distribution of loadings in significant PIB-PET and FDG-PET components


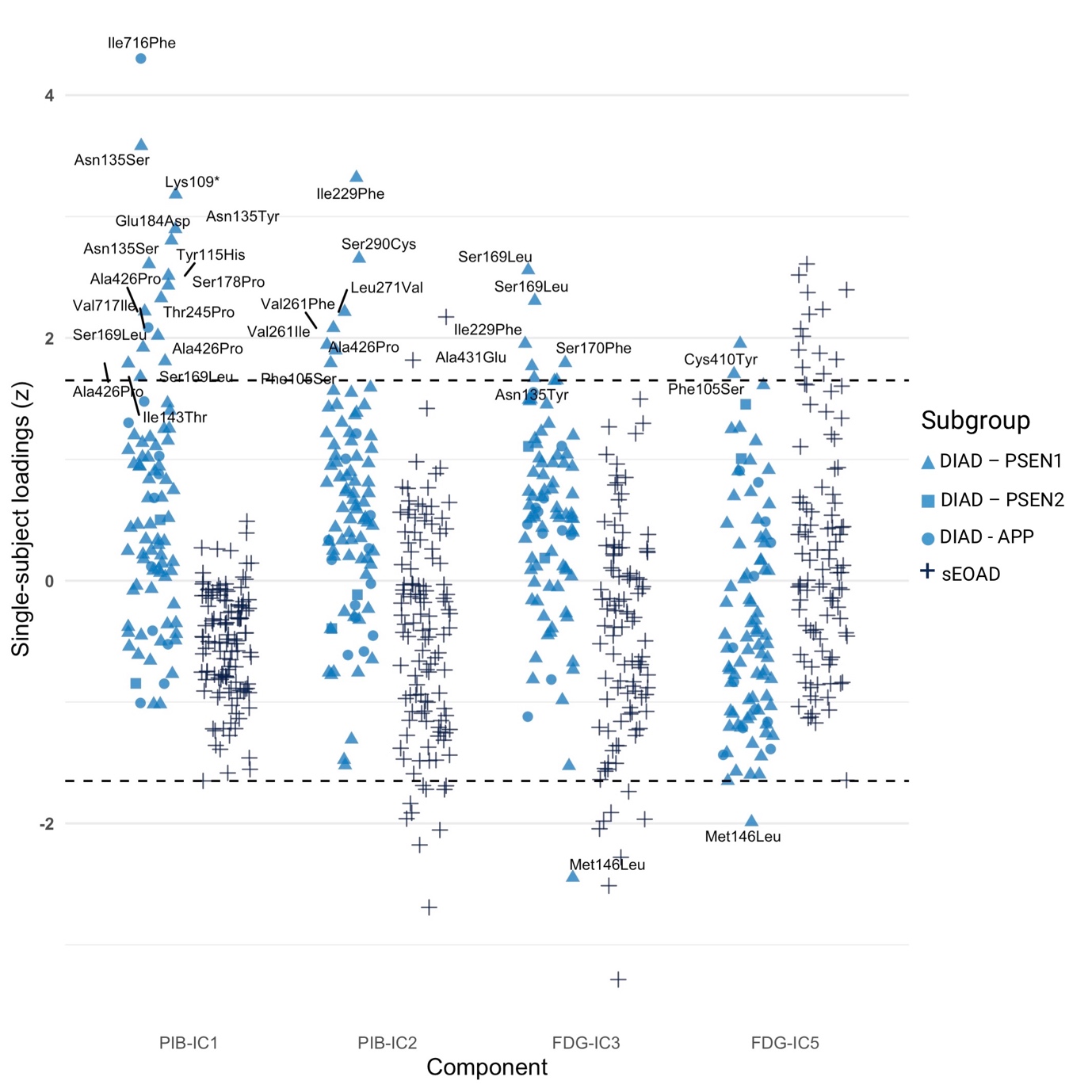


Figure showing distribution of loadings in sEOAD and DIAD participants for all the identified significant PIB-PET and FDG-PET components. Colors and shape index subgroups and dashed lines index whether a loading was |loading|>1.65. DIAD participants with |loading|>1.65 are labeled according to their mutation.

Legend: IC=Independent Component; DIAD=Dominantly Inherited Alzheimer’s Disease; sEOAD= Sporadic Early Onset Alzheimer’s Disease; *PSEN1*=Presinilin-1; *PSEN2*=Presinilin-2; *APP*=Amyloid Precursor Protein

Supplementary Figure 5. Global binding group-level comparisons by target and reference regions in subset of participants (sensitivity analysis)


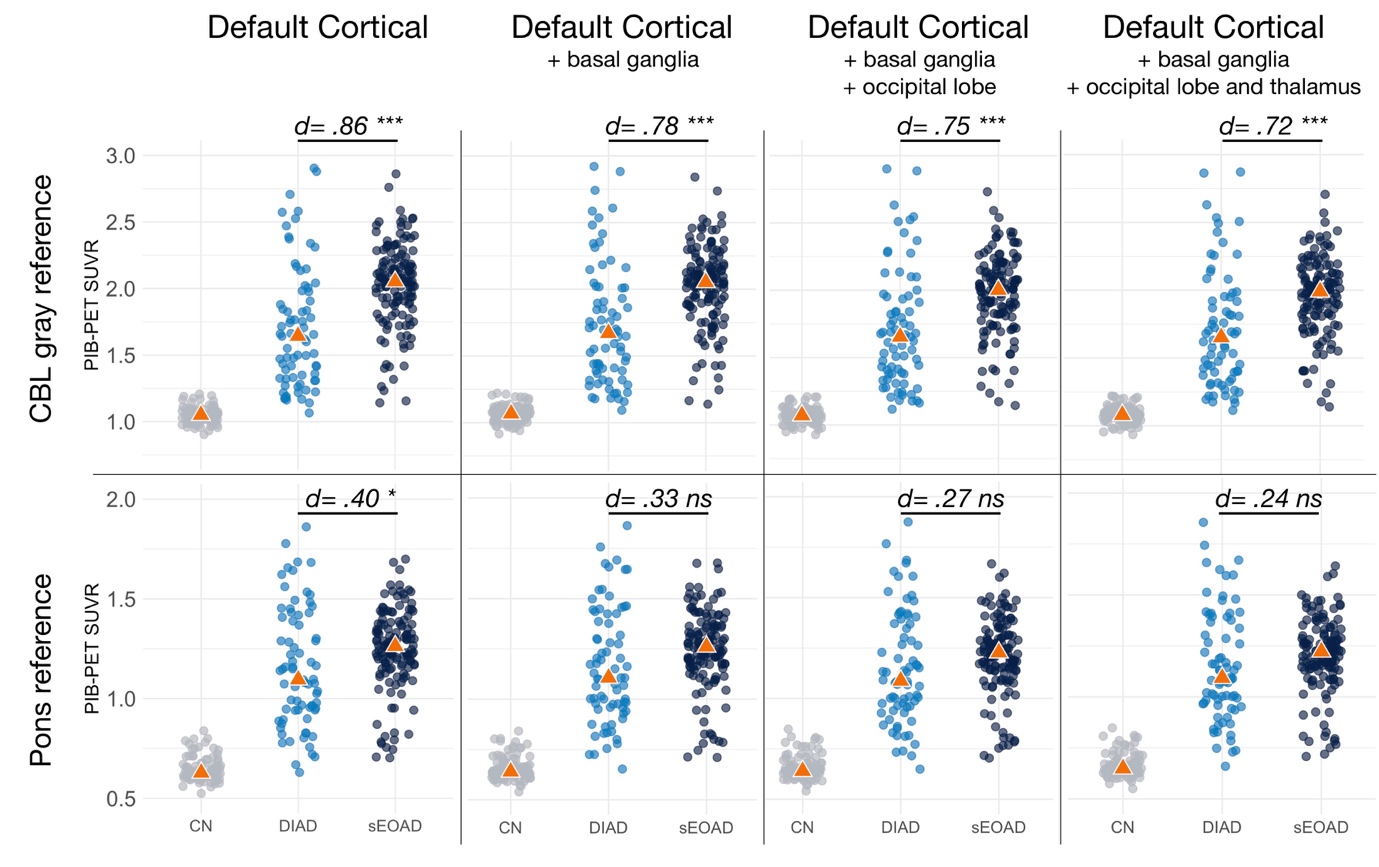


Figure showing distribution of PIB-PET SUVR values according to group, with 8 different combinations of target and reference regions. The default cortical mask used in the study is described in ^52^. This figure is analogous to Supplementary Figure 1, but generated on a subset of participants as sensitivity analysis (see main text for details).

Legend: CBL= Cerebellum; d= Cohen’s d; ***=p<0.001; ** p<0.01; CN=Cognitively Normal; DIAD=Dominantly Inherited Alzheimer’s Disease; sEOAD= Sporadic Early Onset Alzheimer’s Disease

**Supplementary Information on code used for image processing and analysis**

No new software and/or algorithms, nor any specialized or in-house scripts or programs were used for the analyses included in the study.

Image pre-processing and voxelwise analyses were based on standard SPM-based batch processing and functions or other publicly available resources (e.g. DICOM to NIfTI conversion and Freesurfer routines). Statistical analyses and plotting were based on base functions or publicly available R packages. See below for more details.

**Image pre-processing and voxelwise analyses**

**DICOM to NIfTI**: dicm2nii – available at: <https://github.com/xiangruili/dicm2nii>

**PET frames realign**: SPM Realign

**PET frames average**: SPM Util IMcalc

**MRI processing**: Freesurfer recon-all -all + Freesurfer BrainStem module segmentBS.sh

**MRI segmentation**: SPM Segment

**PET co-registration to MRI**: SPM Coregister

**PET SUVr Image creation**: SPM Util IMcalc

**PET SUVr global values extractions**: Image reading and handling with spm_vol() and spm_read_vols()

**PET warping to MNI space**: SPM Util Deformations (applying MRI-based transformation parameters from SPM segment)

**PET Voxelwise Group Comparisons**: SPM Factorial Design Specification: Two Sample t-test with covariates

**PET Transformation of t-maps to effect size maps**: CAT12 (<http://www.neuro.uni-jena.de/cat/>) toolbox – Data Presentation – Threshold and Transform spmT images

**ICA Analysis**: Analyses performed with GIFT toolbox ((<https://trendscenter.org/software/gift/>), see main text for details.

**Statistical analyses and Plotting in R**

**Plotting**: function *geom_point()* with *position_jitterdodge()* and faceting with *facet_wrap()* from package *ggplot2*

**Statistical analyses**

**Descriptive Stats**:

- function *describe* from package *psych*
- base function *table*

**Hypothesis testing**

- function *t.test* from package *stats*
- Cohen D effect size with function *cohen.d* from package *effsize*
- Function *chisq.test* from package *stats*
- Cramer’s V effect size with function *cramerV* from package *rcompanion*
- P-value adjustment with function *p.adjust* from package *stats*
